# Supplementary material for: Picture semantic similarity search based on bipartite network of picture-tag type
Source: PLoS One. 2021 Nov 1;16(11):e0259028. doi: 10.1371/journal.pone.0259028 (PMC8559930; doi:10.1371/journal.pone.0259028)
Supplement: Attachment — Submitted filename: Response to Reviewers.docx [file pone.0259028.s001.docx]

Dear Editors and Reviewers:

Thanks very much for your letter and the comments concerning our manuscript entitled **“Picture Semantic Similarity Search Based on Bipartite Network of Picture-Tag Type” (ID: PONE-D-21-06636).** Those comments are all valuable and very helpful for revising and improving our paper, as well as the important guiding significance to the researches. We have studied comments carefully and have made correction which we hope meet with approval.

The main corrections in the paper and the response to the editor and reviewers are as flowing:

**Journal Requirements：**

We note that Tables 3 and 4 in your submission contain copyrighted images. All PLOS content is published under the Creative Commons Attribution License (CC BY 4.0), which means that the manuscript, images, and Supporting Information files will be freely available online, and any third party is permitted to access, download, copy, distribute, and use these materials in any way, even commercially, with proper attribution. For more information, see our copyright guidelines: http://journals.plos.org/plosone/s/licenses-and-copyright.

We require you to either (1) present written permission from the copyright holder to publish these figures specifically under the CC BY 4.0 license, or (2) remove the figures from your submission:

1. You may seek permission from the original copyright holder of pictures in Tables 3 and 4 to publish the content specifically under the CC BY 4.0 license.

We recommend that you contact the original copyright holder with the Content Permission Form (http://journals.plos.org/plosone/s/file?id=7c09/content-permission-form.pdf) and the following text:

“I request permission for the open-access journal PLOS ONE to publish XXX under the Creative Commons Attribution License (CCAL) CC BY 4.0 (http://creativecommons.org/licenses/by/4.0/). Please be aware that this license allows unrestricted use and distribution, even commercially, by third parties. Please reply and provide explicit written permission to publish XXX under a CC BY license and complete the attached form.”

Please upload the completed Content Permission Form or other proof of granted permissions as an "Other" file with your submission.

In the figure caption of the copyrighted figure, please include the following text: “Reprinted from [ref] under a CC BY license, with permission from [name of publisher], original copyright [original copyright year].”

2. If you are unable to obtain permission from the original copyright holder to publish these figures under the CC BY 4.0 license or if the copyright holder’s requirements are incompatible with the CC BY 4.0 license, please either i) remove the figure or ii) supply a replacement figure that complies with the CC BY 4.0 license. Please check copyright information on all replacement figures and update the figure caption with source information. If applicable, please specify in the figure caption text when a figure is similar but not identical to the original image and is therefore for illustrative purposes only.

**Response:**

After reading the journal requirements carefully, I tried our best to obtain permission from the original copyright holder of the pictures in table 3 and 4. However, the website has not copyright and should be asked permission by the authors. It is difficult to get permission from all authors, so we had to remove table 3 and 4. These changes will not influence the content and framework of the paper.

**Reviewers’ comments:**

[Note: HTML markup is below. Please do not edit.]

Reviewers' comments:

Reviewer's Responses to Questions

Comments to the Author

1. Is the manuscript technically sound, and do the data support the conclusions?

The manuscript must describe a technically sound piece of scientific research with data that supports the conclusions. Experiments must have been conducted rigorously, with appropriate controls, replication, and sample sizes. The conclusions must be drawn appropriately based on the data presented.

Reviewer #1: Yes

Reviewer #2: Yes

2. Has the statistical analysis been performed appropriately and rigorously?

Reviewer #1: Yes

Reviewer #2: I Don't Know

3. Have the authors made all data underlying the findings in their manuscript fully available?

The PLOS Data policy requires authors to make all data underlying the findings described in their manuscript fully available without restriction, with rare exception (please refer to the Data Availability Statement in the manuscript PDF file). The data should be provided as part of the manuscript or its supporting information, or deposited to a public repository. For example, in addition to summary statistics, the data points behind means, medians and variance measures should be available. If there are restrictions on publicly sharing data—e.g. participant privacy or use of data from a third party—those must be specified.

Reviewer #1: Yes

Reviewer #2: Yes

4. Is the manuscript presented in an intelligible fashion and written in standard English?

PLOS ONE does not copyedit accepted manuscripts, so the language in submitted articles must be clear, correct, and unambiguous. Any typographical or grammatical errors should be corrected at revision, so please note any specific errors here.

Reviewer #1: Yes

Reviewer #2: Yes

5. Review Comments to the Author

Please use the space provided to explain your answers to the questions above. You may also include additional comments for the author, including concerns about dual publication, research ethics, or publication ethics. (Please upload your review as an attachment if it exceeds 20,000 characters)

Reviewer #1: 1. What are the limitations of the existing works?

2. The English language has to be polished.

3. Some of the recent works on ML/AI such as the following can be discussed in the paper: "Image-Based malware classification using ensemble of CNN architectures (IMCEC), A Novel PCA-Whale Optimization based Deep Neural Network model for Classification of Tomato Plant Diseases using GPU, Deep learning and medical image processing for coronavirus (COVID-19) pandemic: A survey, Hand gesture classification using a novel CNN-crow search algorithm".

4. Summarize the related works section in the form of a table.

5. Compare the current work with recent state-of-the-art.

6. Present a detailed analysis on the results obtained.

7. Present the computational complexity of the current work.

8. Discuss about the limitations of the current work in conclusion.

Reviewer #2: - Paper is well written. Author should add a little background of the study and limitations of the existing works and clearly explain the contributions at the end of the introduction.

- Qualities of figures are not good.

- Authors should add the most recent reference:

1 Cross corpus multi-lingual speech emotion recognition using ensemble learning, Complex & Intelligent Systems, 1-10

2) Byte-level object identification for forensic investigation of digital images, 2020 International Conference on Cyber Warfare and Security (ICCWS), 1-4

6. If you would like your identity to be revealed to the authors, please include your name here (optional).

Your name and review will not be published with the manuscript.

Reviewer #1: (No Response)

Reviewer #2: (No Response)

**Response to reviewers:**

1. **Response to Reviewer #1:**

**1) Comment:** “What are the limitations of the existing works?”

**Response:** Thanks very much for your comments. We recognized that it is very important to discuss the limitations of existing works. For discussing the limitations, we wrote, “Practically, the measurement of picture similarity should be based on semantic information rather than visual features, which could cause a “semantic gap” between “semantic similarity” by human judgments and “visually similarity” by computer judgments. More precisely, picture semantic similarity is to answer the question “how similar are these two pictures?”. For example, if there are two pictures with different colors and backgrounds in “Cell phone” advertisements, which should be considered to be similar semantically, but they might be treated as dissimilar in visual features. On the other hand, different semantic pictures with similar visual features are judged to be similar pictures by the content-based methods. Due to the lack of semantic consideration, content-based metrics mainly focus on finding similar pictures in terms of visual features rather than semantics, which might neglect the expected similar pictures and deviate from the user’s intention.” **(Line 30-41)**.

**2) Comment:** “The English language has to be polished.”

**Response:** Thanks very much for your valuable comments regarding our paper. By your comments, we realize that the language should be polished, which is important to improve the quality of our manuscript. And we have invited a fluent English speaking colleague who has thoroughly improved the manuscript in terms of the English presentation. The langue was carefully polished, and it is much smoother now. Thank you very much again.

**3) Comment:** “Some of the recent works on ML/AI such as the following can be discussed in the paper: “Image-Based malware classification using ensemble of CNN architectures (IMCEC), A Novel PCA-Whale Optimization based Deep Neural Network model for Classification of Tomato Plant Diseases using GPU, Deep learning and medical image processing for coronavirus (COVID-19) pandemic: A survey, Hand gesture classification using a novel CNN-crow search algorithm”.”

**Response:** Thanks very much for your comments. By your suggestions, we added some discussions in the revised manuscript, “IMCEC [16] employs a deeper architecture of CNNs to provide different semantic representations of the picture, which makes it possible to extract features with higher qualities. [17] proposes a hybrid PCA–whale optimization-based deep learning model for the classification of picture, including transform picture dataset by one-hot encoding approach, reduce the dimensions of the transformed data by PCA and select the optimal features by WOA. [18] discusses the application of DL in medical image processing, which could realize the tracking, diagnosis and treatment of virus spread.” **(Line 19-26)**.

Ref:

16. Danish Vasan SWBSQZ Mamoun Alazab. Image-Based malware classification using ensemble of CNN architectures (IMCEC). Comput Secur. 2020; 92: 101748.

17. Gadekallu T, Rajput D, Reddy P, Lakshman K, Bhattacharya S, Singh S, et al. A novel PCA–whale optimization-based deep neural network model for classification of tomato plant diseases using GPU. Journal of Real-Time Image Processing. 2020 06; p. 1–14.

18. Bhattacharya S, Reddy Maddikunta PK, Pham QV, Gadekallu TR, Krishnan S SR, Chowdhary CL, et al. Deep learning and medical image processing for coronavirus (COVID-19) pandemic: A survey. Sustainable Cities and Society. 2021; 65:102589.

**4) Comment:** “Summarize the related works section in the form of a table.”

**Response:** Thanks for your insightful comments and valuable suggestions. By your comments, we select the most related works to summarize in the form of a table, as show in Table 1, and we wrote, “Table 1 summarizes several picture similarity search methods, including content-based and link-based.” **(Line 80-81)**.

**5) Comment:** “Compare the current work with recent state-of-the-art.”

**Response:** Thanks very much for your comments. After carefully studying your comment, we have compared the current work with recent state-of-the-art in the revised version, as we note, “Compared with the latest content-based metrics, link-based similarity measures could capture the semantic information of pictures based on a picture-tag network, while content-based methods mainly focus on searching similar pictures in visual features, which might neglect the expected similar pictures and deviate from the user’s intention. Moreover, the intuition of link-based methods is that “two pictures are similar if they are related to similar pictures”, which could search underlying similar pictures. For example, picture A is similar to picture B, and picture A is similar to picture C, so picture B is similar to picture C.” **(Line 81-88)**.

**6) Comment:** “Present a detailed analysis on the results obtained.”

**Response:** Thanks very much for your comments. By your comments, we realize that it is important to analysis the results in detail. Correspondingly, we made more thorough discussions regarding the experimental results, as we wrote, “Table 2 shows the MAP scores of different metrics in Nicpic, and PictureSim sets l as 5. The MAP scores of PictureSim are obviously higher than that of traditional content-based methods with different k. For example at k = 15, PictureSim achieves average 0.599 MAE, while RD and HI yield average 0.119 MAE. This is because PictureSim computes similarity scores by the structure of context in the picture-tag network, while the traditional content-based approach considers the visual features, which often fails to reflect the semantic information in the user’s mind.” **(Line 270-276)**, “Fig. 2(a) shows the MAP scores with varying l in Nipic, which clearly illustrates the effect of l in PictureSim. We observe that the MAP scores increase slowly as l increases from 1 to 5, because PictureSim not only considers direct in-links among nodes but also indirect in-links. After l = 5, the MAP scores become stable, and PictureSim converges to a stable state. So, the returned rankings would become stable empirically after the fifth iteration.” **(Line 277-282)**, “Fig. 2(b) shows the MAP scores of PictureSim on varying k in Nipic. The MAP scores gradually decrease as k increases; it could achieve average 0.718 MAP at k = 5. This is because the higher similarity scores have a higher rank in the returned list. Generally, users are only interested in the top 10 similar pictures for a given picture, so PictureSim could achieve the user’s intention.” **(Line 283-287)**, “Fig. 4(a) shows the running time on varying l in Nipic, in which, the running time increases slowly before l = 5 and increases rapidly after l = 5. This is because PictureSim also considers indirect in-links when searching similar pictures, it needs to traverse more paths as l increases. Fortunately, PictureSim could converge rapidly at l = 5 as shown in Fig. 2(a), which shows a good performance of the proposed approach.” **(Line 307-312)**, “Fig. 4(b) shows the running time on varying k in Nipic, where l =1, 3, 5, 7. We observe that the running time almost remains stable as k increases, which indicates time overhead does not change as k increases. This is because running time is affected by the sorting rather than the similarity calculation, and sorting overhead is almost negligible compared with the computational overhead. And the running time fluctuates significantly at l = 7, due to the instability of the machine.” **(Line 313-318)**, “Fig. 5(a) shows the running time on varying h in Nipic. We observe that the running time decreases as h increases from 0.1 to 0.9. It drops rapidly from h = 0 to 0.6, and afterward, the slowly decreases as h increases. Because a larger h, the more noisy links will be removed in picture-tag network, which indicates the efficiency can be significantly improved after h = 0.6. So, we set h as 0.8 if not specified in other experiments.” **(Line 319-324)**, “Fig. 6(a) shows the MAP scores of PictureSim on varying l in ImageNet. We observe that the MAP scores of PictureSim is relatively lower than that of Nipic. The reason is that each picture is described by only one tag, which fails to fully express the semantics information of the picture. Moreover, MAP scores irregularly fluctuate as l increases, including the ranking of top 5, top 10 and top 15, because PictureSim searches all similar pictures at l = 1 and it has same similarity scores. However, the returned ranking is different due to the sort algorithm. Fig. 6(b) shows the MAP scores of PictureSim on varying k in ImageNet. The results are similar to Fig. 2(b), but the curve relatively fluctuates compared with Fig. 2(b), and the difference between maximum and minimum is smaller than Nipic, the reason is as mentioned above.” **(Line 332-341)**, and “Fig. 7(a) shows the running time on varying l in ImageNet, where k =5, 10, 15. In which, the running time increases as l increases. But the time overhead is very small, especially ImageNet takes 0.004s at l = 7, while Nipic needs 7.3s. This is because each picture is described by only one tag, the picture-tag network is very sparse in ImageNet. Fig. 7(b) shows the running time on varying k in ImageNet, where l =1, 3, 5, 7. The result is similar to Fig. 4(b), and the reason is as mentioned above. But the fluctuate of curve is relatively evident compared with Nipic. Because the time overhead of ImageNet is very small, so the time overhead of sort is relatively evident.” **(Line 342-349)**.

**7) Comment:** “Present the computational complexity of the current work.”

**Response:** Thanks very much for your comments. In our manuscript, we first analyzed the computational complexity of SimRank, as we wrote, “Though this can be saved time cost in on-line stage, expensive operations are required in the off-line stage, which involves O(n^2^) time cost and O(ld^2^n^2^) space cost at the l-th iteration, where n is the number of nodes in the network, d is the average degree of the nodes, and we set l from 1 to 7 in terms of time and cost overhead.” **(Line 224-228)**. And then we analyzed the computational complexity of the PictureSim, as we wrote, “Therefore, PictureSim optimizes the efficiency of SimRank by TopSim algorithm without any preprocessing, which requires O(d^2l^) time cost in the on-line stage.” **(Line 235-237)**.

**8) Comment:** “Discuss about the limitations of the current work in conclusion.”

**Response:** Thanks very much for your comments. After checking the paper carefully, we recognize that it is necessary to discuss the limitations of the current work, and we wrote, “Future work will extend our approach to other datasets for effectively searching similar objects in other fields, because PictureSim is proposed for searching semantically similar pictures. Then, PictureSim requires O(d^2l^) time cost, and the number of paths increases exponentially as path length increases, which makes computation expensive in terms of time and space and cannot support fast similarity search over large networks. So, we will focus on reducing computational overhead to ensure timely response in large networks.” **(Line 369-375)**.

**Response to Reviewer #2:**

**1) Comment:** “Paper is well written. Author should add a little background of the study and limitations of the existing works and clearly explain the contributions at the end of the introduction.”

**Response:** Thanks for your insightful comments. By your comments, the background and limitations of the existing works are discussed, as we wrote, “Practically, the measurement of picture similarity should be based on semantic information rather than visual features, which could cause a “semantic gap” between “semantic similarity” by human judgments and “visually similarity” by computer judgments. More precisely, picture semantic similarity is to answer the question “how similar are these two pictures?”. For example, if there are two pictures with different colors and backgrounds in “Cell phone” advertisements, which should be considered to be similar semantically, but they might be treated as dissimilar in visual features. On the other hand, different semantic pictures with similar visual features are judged to be similar pictures by the content-based methods. Due to the lack of semantic consideration, content-based metrics mainly focus on finding similar pictures in terms of visual features rather than semantics, which might neglect the expected similar pictures and deviate from the user’s intention.” **(Line 30-41)**. Then, we explain the contributions of our works more clearly, as we wrote in the introduction, “We build a picture-tag network by “description” relationships between pictures and tags. Initially, tags and pictures are treated as nodes, and relationships between pictures and tags are regarded as edges. Then, we propose a TF-IDF-based method to remove the noisy links by setting a threshold, which could measure whether a tag has good classification performance. We propose a link-based picture similarity search algorithm, namely PictureSim, for effectively searching similar pictures semantically, which considers the context structure to search underlying similar pictures in a network. And it could respond to the user’s requirement timely. We ran a comprehensive set of experiments on Nipic datasets and ImageNet datasets. Our results show that PictureSim achieves semantic similarity search between pictures, which produces a better correlation with human judgments compared with content-based methods.” **(Line 99-111)**.

**2) Comment:** “Qualities of figures are not good.”

**Response:** Thanks very much for your valuable comments. Inspired by your suggestions, we improve the qualities of figures, such as the font size, icon size and so on. Figure 2 is example.

**3) Comment:** “Authors should add the most recent reference:

1) Cross corpus multi-lingual speech emotion recognition using ensemble learning, Complex & Intelligent Systems, 1-10

2) Byte-level object identification for forensic investigation of digital images, 2020 International Conference on Cyber Warfare and Security (ICCWS), 1-4”

**Response:** Thanks very much for your valuable comments, we have read the most recent reference carefully. Inspired by your suggestions, we added some references, as we wrote “[19] proposes an effective ensemble learning approach to identify and detect objects, which could achieve good accuracy on both with-in as well as cross-corpus datasets. [20] proposes a deep learning-based object detection approach, which utilizes ResNet to achieve fast robust and efficient object detection.” **(Line 26-29)**.

Ref:

19. Zehra W, Javed AR, Jalil Z, Gadekallu T, Kahn H. Cross corpus multi-lingual speech emotion recognition using ensemble learning. Complex & Intelligent Systems. 2021 01; p. 1–10.

20. Javed AR, Jalil Z. Byte-Level Object Identification for Forensic Investigation of Digital Images; 2020. p. 1–4.

We tried our best to improve the manuscript and made some changes in the manuscript. These changes will not influence the content and framework of the paper.

We appreciate for your warm work earnestly, and hope that the correction will meet with approval.

Once again, thank you very much for your comments and suggestions.

Yours sincerely,

Mingxi Zhang
