# Supplementary material for: Picture semantic similarity search based on bipartite network of picture-tag type
Source: PLoS One. 2021 Nov 1;16(11):e0259028. doi: 10.1371/journal.pone.0259028 (PMC8559930; doi:10.1371/journal.pone.0259028)
Supplement: Attachment — Submitted filename: Response to Reviewers.docx [file pone.0259028.s002.docx]

Dear Editors and Reviewers:

Thanks very much for your letter and the comments concerning our manuscript entitled **“Picture Semantic Similarity Search Based on Bipartite Network of Picture-Tag Type” (ID: PONE-D-21-06636R1).** Those comments are all valuable and very helpful for revising and improving our paper, as well as the important guiding significance to the researches. We have studied comments carefully and have made correction which we hope meet with approval.

The main corrections in the paper and the response to the editor are as flowing:

**Journal Requirements：**

Please review your reference list to ensure that it is complete and correct. If you have cited papers that have been retracted, please include the rationale for doing so in the manuscript text, or remove these references and replace them with relevant current references. Any changes to the reference list should be mentioned in the rebuttal letter that accompanies your revised manuscript. If you need to cite a retracted article, indicate the article’s retracted status in the References list and also include a citation and full reference for the retraction notice.

**Response:**

After reading the journal requirements, we carefully reviewed the cited papers in our manuscript. And we did not find any paper that has been retracted in the reference list. Thanks for your suggestions.

**Reviewers’ comments:**

[Note: HTML markup is below. Please do not edit.]

Reviewers' comments:

Reviewer's Responses to Questions

Comments to the Author

1. Is the manuscript technically sound, and do the data support the conclusions?

The manuscript must describe a technically sound piece of scientific research with data that supports the conclusions. Experiments must have been conducted rigorously, with appropriate controls, replication, and sample sizes. The conclusions must be drawn appropriately based on the data presented.

Reviewer #1: Yes

Reviewer #2: Yes

2. Has the statistical analysis been performed appropriately and rigorously?

Reviewer #1: Yes

Reviewer #2: I Don't Know

3. Have the authors made all data underlying the findings in their manuscript fully available?

The PLOS Data policy requires authors to make all data underlying the findings described in their manuscript fully available without restriction, with rare exception (please refer to the Data Availability Statement in the manuscript PDF file). The data should be provided as part of the manuscript or its supporting information, or deposited to a public repository. For example, in addition to summary statistics, the data points behind means, medians and variance measures should be available. If there are restrictions on publicly sharing data—e.g. participant privacy or use of data from a third party—those must be specified.

Reviewer #1: Yes

Reviewer #2: Yes

4. Is the manuscript presented in an intelligible fashion and written in standard English?

PLOS ONE does not copyedit accepted manuscripts, so the language in submitted articles must be clear, correct, and unambiguous. Any typographical or grammatical errors should be corrected at revision, so please note any specific errors here.

Reviewer #1: Yes

Reviewer #2: Yes

5. Review Comments to the Author

Please use the space provided to explain your answers to the questions above. You may also include additional comments for the author, including concerns about dual publication, research ethics, or publication ethics. (Please upload your review as an attachment if it exceeds 20,000 characters)

Reviewer #1: 1. What are the limitations of the existing works?

2. The English language has to be polished.

3. Some of the recent works on ML/AI such as the following can be discussed in the paper: "Image-Based malware classification using ensemble of CNN architectures (IMCEC), A Novel PCA-Whale Optimization based Deep Neural Network model for Classification of Tomato Plant Diseases using GPU, Deep learning and medical image processing for coronavirus (COVID-19) pandemic: A survey, Hand gesture classification using a novel CNN-crow search algorithm".

4. Summarize the related works section in the form of a table.

5. Compare the current work with recent state-of-the-art.

6. Present a detailed analysis on the results obtained.

7. Present the computational complexity of the current work.

8. Discuss about the limitations of the current work in conclusion.

Reviewer #2: - Paper is well written. Author should add a little background of the study and limitations of the existing works and clearly explain the contributions at the end of the introduction.

- Qualities of figures are not good.

- Authors should add the most recent reference:

1 Cross corpus multi-lingual speech emotion recognition using ensemble learning, Complex & Intelligent Systems, 1-10

2) Byte-level object identification for forensic investigation of digital images, 2020 International Conference on Cyber Warfare and Security (ICCWS), 1-4

6. If you would like your identity to be revealed to the authors, please include your name here (optional).

Your name and review will not be published with the manuscript.

Reviewer #1: (No Response)

Reviewer #2: (No Response)

**Response to Editor:**

1. **Response to minor misprints:**

**1) Comment:**

- "Content-based Image Retrieval*(CBIR)*" (there is a missing space)

- "they point to similar objects*.*."

- "Oriol et al. [38] *proposes*"

- "As *show* in Fig. 1,"

- "where c *ia* a constant"

- "Term frequency*(TF)*"

- "inverse document frequency*(IDF)*"

- "Minkowski Distance*(MD)* [42], Histogram Intersection*(HI)* [43] and Relative Deviation*(RD)*"

- "by Mean Average Precision*(MAP)*"

Additional Editor Comments (if provided):

Minor misprints:

**Response:** Thanks very much for your comments. We have revised these misprints and carefully checked the manuscript. Specifically, “Content-based Image Retrieval(CBIR)” was corrected to “Content-based Image Retrieval (CBIR)” **(Line 6-7)**, in which the missing space was added; “they point to similar objects..” was corrected to “they point to similar objects.” **(Line 51-52)**, in which symbol “.” was deleted; “Oriol et al. [38] proposes” was changed to “Oriol et al. [38] proposed” **(Line 138-139)**; “As show in Fig. 1,” was changed to “As shown in Fig. 1,” **(Line 198);** “where c ia a constant” was changed to “where c is a constant” **(Line 208);** “Term frequency(TF)” was changed to “Term frequency (TF)” **(Line 169-170)** by adding a space before “(TF)”; “inverse document frequency(IDF)” was changed to “inverse document frequency (IDF)” **(Line 174-175)** by adding a space before “(IDF)”; “Minkowski Distance(MD)[42], Histogram Intersection(HI)[43] and Relative Deviation(RD)” was changed to “Minkowski Distance (MD) [42], Histogram Intersection (HI) [43] and Relative Deviation (RD) ” **(Line 251-252)** by adding spaces in the corresponding spaces; and “by Mean Average Precision(MAP)” was corrected to “by Mean Average Precision (MAP)” by adding spaces before “(MAP)” **(Line 261-262)**.

Thanks again for your valuable suggestions.

We tried our best to improve the manuscript and made some changes in the manuscript. These changes will not influence the content and framework of the paper.

We appreciate for your warm work earnestly, and hope that the correction will meet with approval.

Once again, thank you very much for your comments and suggestions.

Yours sincerely,

Mingxi Zhang
